# Non-invasive Evaluation of Myocardial Fibrosis Using T1 and T2 Mapping by Cardiac Magnetic Resonance Imaging

**DOI:** 10.7759/cureus.105441

**Published:** 2026-03-18

**Authors:** Maynor Jose Lopez Mendoza, Nicolle Contreras Figueroa, Maria Antonieta Salazar Estrada, Jeilyn Jiron Vindas, Asdrubal Ulloa, María Jennifer Valle Mena

**Affiliations:** 1 Anesthesiology and Perioperative Medicine, Hospital de las Mujeres Dr. Adolfo Carit Eva (CARITEVA), San José, CRI; 2 General Medicine, Caja Costarricense de Seguro Social (CCSS), San José, CRI; 3 Emergency, Hospital Los Chiles, Los Chiles, CRI; 4 Obstetrics and Gynecology, Hospital de las Mujeres Dr. Adolfo Carit Eva (HOMACE), San José, CRI; 5 Gynecologic Oncology, Hospital de las Mujeres Dr. Adolfo Carit Eva (HOMACE), San José, CRI; 6 General Medicine, Área de Salud Upala, Alajuela, CRI

**Keywords:** cardiac magnetic resonance, extracellular volume, myocardial fibrosis, risk stratification, t1 mapping, t2 mapping

## Abstract

Myocardial fibrosis is a common pathological substrate across a broad spectrum of cardiovascular diseases and represents a key determinant of adverse clinical outcomes, including heart failure progression, arrhythmias, and sudden cardiac death. It may present as focal replacement fibrosis, typically following myocardial infarction, or as diffuse interstitial fibrosis, more frequently observed in non-ischemic cardiomyopathies and pressure-overload conditions. At the cellular level, fibrosis is driven by fibroblast activation and differentiation into myofibroblasts, mediated by profibrotic signaling pathways and sustained extracellular matrix deposition. Inflammation and myocardial edema also play a critical role in initiating and amplifying fibrotic remodeling, linking acute injury to chronic structural changes.

Cardiac magnetic resonance imaging has emerged as a reference non-invasive modality for myocardial tissue characterization. Parametric mapping techniques, including T1, T2, and extracellular volume fraction mapping, provide a quantitative and highly reproducible assessment of myocardial composition. Native T1 mapping and extracellular volume fraction allow sensitive detection and quantification of diffuse myocardial fibrosis and have demonstrated significant prognostic value across several cardiovascular conditions, while T2 mapping is particularly sensitive to myocardial edema and active inflammation, enabling differentiation between acute and chronic myocardial injury. When integrated, these techniques enable a comprehensive evaluation of the inflammation-fibrosis continuum.

Clinically, T1 and T2 mapping have demonstrated substantial value across ischemic and non-ischemic heart diseases, valvular disorders, and infiltrative cardiomyopathies. These techniques contribute to improved risk stratification, prediction of adverse ventricular remodeling, guidance of therapeutic decision-making, and monitoring of treatment response. Compared with late gadolinium enhancement imaging, echocardiographic strain analysis, and circulating biomarkers, parametric mapping offers greater tissue specificity and quantitative characterization of myocardial remodeling, although its implementation requires specialized equipment and technical expertise.

## Introduction and background

Myocardial fibrosis represents a common pathological endpoint in a wide spectrum of cardiovascular diseases and has been consistently associated with adverse clinical outcomes, including the development of heart failure, ventricular arrhythmias, and an increased risk of sudden cardiac death. Fibrotic remodeling is observed across several major cardiovascular conditions, including ischemic heart disease, hypertensive heart disease, cardiomyopathies, and valvular disorders. Epidemiological and imaging studies suggest that myocardial fibrosis may be present in up to 40%-60% of patients with chronic heart failure and in a substantial proportion of individuals with cardiomyopathies, highlighting its central role in myocardial remodeling and disease progression. This process arises from the excessive accumulation of extracellular matrix components within the myocardial interstitium, which progressively alters normal tissue architecture and impairs both the mechanical and electrical functions of the heart [1,2]. As fibrotic remodeling advances, it creates a structural and electrophysiological substrate that promotes ventricular dysfunction and electrical instability, thereby facilitating the occurrence of arrhythmias. Consequently, early identification and characterization of myocardial fibrosis are of central importance for timely intervention and effective clinical management [3].

Despite its diagnostic value, endomyocardial biopsy presents important limitations in routine clinical practice. Although it remains the definitive method for histological confirmation, its invasive nature exposes patients to procedural risks, including bleeding and infection. Moreover, because myocardial fibrosis often exhibits a focal or heterogeneous distribution, biopsy samples obtained from limited myocardial regions may fail to accurately reflect the true extent of fibrotic involvement, resulting in substantial sampling error [4]. Alternative indirect diagnostic approaches, such as circulating laboratory biomarkers, have also been explored; however, these methods generally lack the specificity and sensitivity required for precise detection and quantification of myocardial fibrosis [1].

In this context, cardiac magnetic resonance imaging has emerged as a reference noninvasive modality for myocardial tissue characterization. Its ability to generate high-resolution images of cardiac anatomy and function enables detailed evaluation of myocardial composition and structural remodeling [1,4]. Importantly, cardiac magnetic resonance imaging allows assessment of both focal and diffuse forms of myocardial fibrosis, providing valuable information regarding the extent, distribution, and severity of fibrotic remodeling [5]. In addition, this imaging modality enables longitudinal evaluation, facilitating monitoring of disease progression and therapeutic response over time, which further enhances its clinical utility in patient management [6].

Within cardiac magnetic resonance imaging, parametric mapping techniques have gained increasing attention due to their quantitative nature and diagnostic versatility. T1 mapping refers to the measurement of the longitudinal relaxation time of myocardial tissue and provides quantitative information about tissue composition, including estimation of extracellular volume fraction, a parameter closely associated with the burden of diffuse myocardial fibrosis [7]. In contrast, T2 mapping measures the transverse relaxation time and is particularly sensitive to variations in myocardial water content, enabling detection of myocardial edema and inflammatory activity. Because inflammatory injury and edema frequently precede or accompany fibrotic remodeling, these techniques provide complementary insights into the continuum between myocardial injury, inflammation, and fibrosis. Furthermore, T1 and T2 mapping approaches have been extensively validated across diverse clinical scenarios, demonstrating robust performance in the diagnosis, phenotypic characterization, and risk stratification of myocardial diseases [8,9].

The objective of this narrative review is to analyze and synthesize the current evidence regarding the non-invasive evaluation of myocardial fibrosis using T1 and T2 mapping techniques in cardiac magnetic resonance imaging.

## Review

Methods

The present narrative review was developed using a structured methodological framework aimed at synthesizing contemporary evidence on the non-invasive evaluation of myocardial fibrosis using T1 and T2 mapping techniques in cardiac magnetic resonance imaging. The objective was to integrate technical principles, validation studies, and clinical applications of parametric mapping for the assessment of myocardial tissue remodeling across diverse cardiovascular conditions.

A literature search was conducted in PubMed, Scopus, and ScienceDirect. The search primarily focused on studies published between January 2020 and December 2025 in English or Spanish to capture the most recent methodological developments, technological improvements in mapping techniques, and updated clinical evidence. Earlier landmark studies were also considered when they provided foundational contributions to myocardial tissue characterization or the development and validation of cardiac magnetic resonance mapping methodologies.

The search strategy combined Medical Subject Headings and free-text terms using Boolean operators. In PubMed, the following structure was applied: (“myocardial fibrosis”[MeSH] OR “diffuse fibrosis”) AND (“cardiac magnetic resonance” OR “CMR”) AND (“T1 mapping” OR “T2 mapping” OR “extracellular volume” OR “parametric mapping” OR “myocardial edema”). Filters for human studies were applied when available. Equivalent adaptations of this strategy were implemented in the remaining databases.

The initial search yielded 768 records. After removal of duplicates, 642 articles underwent title and abstract screening. Of these, 124 were selected for full-text evaluation, and 71 studies were ultimately included in the final synthesis based on relevance to the objectives of the review and methodological robustness.

Study selection was performed independently by two authors. Discrepancies regarding eligibility were resolved through discussion and consensus. Because this work was designed as a narrative review rather than a formal systematic review, PRISMA-style reporting and flow diagrams were not applied. Nevertheless, study quality was assessed qualitatively, considering aspects such as study design, sample size, the reproducibility of mapping protocols, the standardization of acquisition parameters, and the clarity of reported clinical outcomes.

Inclusion criteria comprised original research studies, technical validation studies, systematic reviews, meta-analyses, and position statements addressing myocardial fibrosis assessment through T1 mapping, T2 mapping, or extracellular volume quantification. Exclusion criteria included duplicate publications, studies without clinical applicability, purely experimental basic science research without translational relevance, and investigations conducted in populations or contexts that are not generalizable to clinical cardiovascular practice.

Given the heterogeneity of mapping protocols, magnetic field strengths, post-processing software, and studied populations, a narrative synthesis was considered more appropriate than a quantitative meta-analytic approach. The final analysis was organized into thematic domains, including the technical principles of T1 and T2 mapping, extracellular volume quantification, the differentiation between fibrosis and inflammation, disease-specific applications, prognostic implications, and current limitations.

Artificial intelligence tools were used exclusively for structural organization and linguistic refinement. All interpretative analyses, study selection, and scientific judgments were performed independently by the authors to ensure academic integrity and methodological rigor.

Pathophysiological basis of myocardial fibrosis

Myocardial fibrosis can be classified into focal and diffuse forms according to its distribution and underlying pathophysiological mechanisms. Focal fibrosis occurs in localized regions of the myocardium and most commonly develops as a consequence of myocardial infarction, where irreversible cardiomyocyte death triggers their replacement with fibrotic tissue. This pattern is therefore frequently referred to as replacement fibrosis and reflects a localized reparative response to ischemic injury. In contrast, diffuse myocardial fibrosis is characterized by widespread involvement of the interstitial and perivascular spaces and is typically observed in non-ischemic cardiac diseases. Rather than forming discrete scars, this form of fibrosis presents a more homogeneous distribution throughout the myocardium, leading to global alterations in myocardial structure and compliance [2,10,11].

At the cellular and molecular level, myocardial fibrosis is driven primarily by the activation of cardiac fibroblasts. Under pathological stimuli, these cells differentiate into myofibroblasts, which represent the main effector cells responsible for fibrotic remodeling. This phenotypic transformation is regulated by multiple signaling pathways, including transforming growth factor-beta with downstream SMAD3 signaling, p38 mitogen-activated protein kinase, and Wnt-beta-catenin signaling, all of which promote fibroblast activation and persistence [12,13]. In addition, several neurohormonal and inflammatory mediators play a central role in fibrotic signaling. Among these, angiotensin II and aldosterone contribute to fibroblast activation and extracellular matrix deposition, while connective tissue growth factor and proinflammatory cytokines further amplify profibrotic signaling pathways within the myocardium.

Once activated, myofibroblasts markedly increase the synthesis of extracellular matrix components, particularly collagen types I and III. The excessive deposition of these structural proteins results in increased myocardial stiffness, impaired diastolic relaxation, and progressive deterioration of cardiac function [14,15]. At the same time, normal extracellular matrix turnover becomes disrupted due to an imbalance between matrix synthesis and degradation. This process involves matrix metalloproteinases, which are responsible for extracellular matrix degradation, and their endogenous regulators, the tissue inhibitors of metalloproteinases. Dysregulation of this matrix metalloproteinase-tissue inhibitor system contributes to excessive extracellular matrix accumulation and the progression of myocardial fibrosis [10].

Inflammation plays a central role in both the initiation and progression of myocardial fibrosis, serving as a critical link between tissue injury and fibrotic remodeling. Inflammatory cells infiltrating the myocardium release a wide range of cytokines and growth factors that stimulate fibroblast activation and enhance extracellular matrix deposition. In this context, myocardial edema frequently accompanies acute inflammatory responses and represents an important intermediate stage in the fibrotic cascade. By increasing tissue pressure and altering the myocardial microenvironment, edema can further amplify fibroblast activation and accelerate the transition from inflammation to fibrosis [16].

Beyond structural remodeling, myocardial fibrosis also has important electrophysiological consequences. The accumulation of fibrotic tissue disrupts normal myocardial architecture and interferes with electrical impulse propagation, leading to heterogeneous conduction and the formation of arrhythmogenic substrates. These alterations contribute to the development of ventricular arrhythmias and represent an important mechanism underlying the increased risk of sudden cardiac death observed in patients with advanced fibrotic remodeling.

The prognostic implications of myocardial fibrosis vary according to the underlying etiology of cardiac disease. In ischemic heart disease, particularly following myocardial infarction, fibrosis initially represents a compensatory response aimed at preserving structural integrity after tissue necrosis. However, when excessive or persistent, this process contributes to adverse ventricular remodeling, progressive systolic dysfunction, and the development of heart failure [14,17]. In non-ischemic heart diseases, such as hypertrophic cardiomyopathy and aortic stenosis, diffuse myocardial fibrosis is a prominent pathological feature. In these conditions, fibrotic remodeling is strongly associated with unfavorable clinical outcomes, including impaired ventricular performance and an increased risk of sudden cardiac death, which underscores its relevance as a marker of disease severity and prognosis [11,17].

Physical principles of cardiac magnetic resonance imaging

The physiological basis of T2 relaxation is closely related to the presence and mobility of water molecules within myocardial tissue. In magnetic resonance imaging, T2 relaxation reflects the decay of transverse magnetization caused by spin-spin interactions between neighboring hydrogen nuclei. Under normal physiological conditions, myocardial water content is tightly regulated. However, in pathological states such as edema and inflammation, the proportion of free water increases, prolonging T2 relaxation times. Consequently, elevated T2 values serve as a sensitive quantitative marker of increased myocardial water content, and are widely interpreted as an imaging surrogate for myocardial edema [8].

In clinical practice, T2 mapping has proven particularly effective for the detection of myocardial edema and inflammatory activity. Myocardial edema often represents one of the earliest manifestations of myocardial injury, and may appear before the onset of clinical symptoms or the development of irreversible structural remodeling [8]. In inflammatory cardiomyopathies, such as myocarditis, as well as in hypertrophic cardiomyopathy, increased T2 values have been shown to correlate with active myocardial inflammation. Importantly, these elevated values are not only diagnostically relevant but also carry prognostic significance, as they have been associated with adverse outcomes, including an increased risk of ventricular arrhythmias [18,19].

Beyond detection, T2 mapping plays a critical role in differentiating between acute and chronic myocardial alterations. Acute myocardial injury is typically characterized by prominent edema, which manifests as increased T2 relaxation times. In contrast, chronic fibrotic remodeling generally exhibits normal or only mildly altered T2 values, while showing abnormalities on T1 mapping and extracellular volume fraction measurements, reflecting collagen deposition rather than active inflammation [20]. This distinction is particularly valuable in systemic and infiltrative diseases. For instance, in Anderson-Fabry disease, T2 mapping allows the identification of ongoing myocardial injury, which has been linked to subsequent scar progression and worsening myocardial involvement [21].

It is important to recognize that quantitative T2 measurements may be influenced by several technical factors. Variations in magnetic field strength, sequence selection, motion artifacts related to cardiac or respiratory motion, susceptibility effects, and partial volume contamination can affect T2 estimation and contribute to measurement variability. In addition, differences in acquisition protocols and scanner vendors may lead to inter-scanner variability, which should be considered when comparing results across institutions or multicenter studies.

The integration of T2 mapping with T1 and extracellular volume mapping further enhances myocardial tissue characterization. When used in combination, these techniques provide a comprehensive assessment of myocardial composition, enabling differentiation among fibrosis, edema, and fat infiltration. This multiparametric approach facilitates a more nuanced understanding of the inflammation-fibrosis continuum, and is especially relevant in complex cardiomyopathies, such as Duchenne cardiomyopathy, where overlapping pathological processes coexist and evolve over time [20].

Myocardial T1 mapping

Native T1 mapping is a quantitative cardiac magnetic resonance technique that measures the intrinsic T1 relaxation time of myocardial tissue without the administration of contrast agents. This parameter reflects fundamental tissue properties and is influenced by myocardial water content, interstitial fibrosis, and infiltrative processes. Baseline native T1 values are not fixed and may vary according to physiological determinants, such as age, sex, and cardiovascular risk factors, which can introduce variability even in the absence of overt disease [22,23].

Alterations in native T1 values are closely associated with underlying myocardial pathology. Elevated native T1 times have been consistently linked to diffuse interstitial fibrosis, myocardial edema, and infiltrative disorders. Such increases are commonly observed in conditions such as cardiac amyloidosis and myocarditis, where expansion of the extracellular space, or increased myocardial water content, leads to prolongation of T1 relaxation times. As a result, changes in native T1 serve as sensitive indicators of subclinical myocardial involvement and may reflect ongoing pathological processes, even before irreversible structural damage becomes apparent [23,24].

Both technical and biological factors significantly influence native T1 measurements, and must be carefully considered in clinical and research settings. Variations in magnetic field strength, particularly between 1.5-tesla and 3-tesla scanners, can lead to systematic differences in absolute T1 values. In addition, the choice of acquisition sequence, such as Modified Look-Locker Inversion recovery, or shortened Modified Look-Locker Inversion recovery, affects measurement accuracy and reproducibility. Beyond technical considerations, several biological conditions may confound the interpretation of native T1 values. For example, anemia may increase T1 values due to changes in blood composition, whereas myocardial lipid accumulation or iron overload may lead to T1 shortening. Systemic inflammatory states may also influence myocardial water content and contribute to variations in measured T1 values. These factors highlight the importance of interpreting native T1 measurements within the broader clinical context, and using scanner- and sequence-specific reference ranges [22,24].

**Figure 1:**
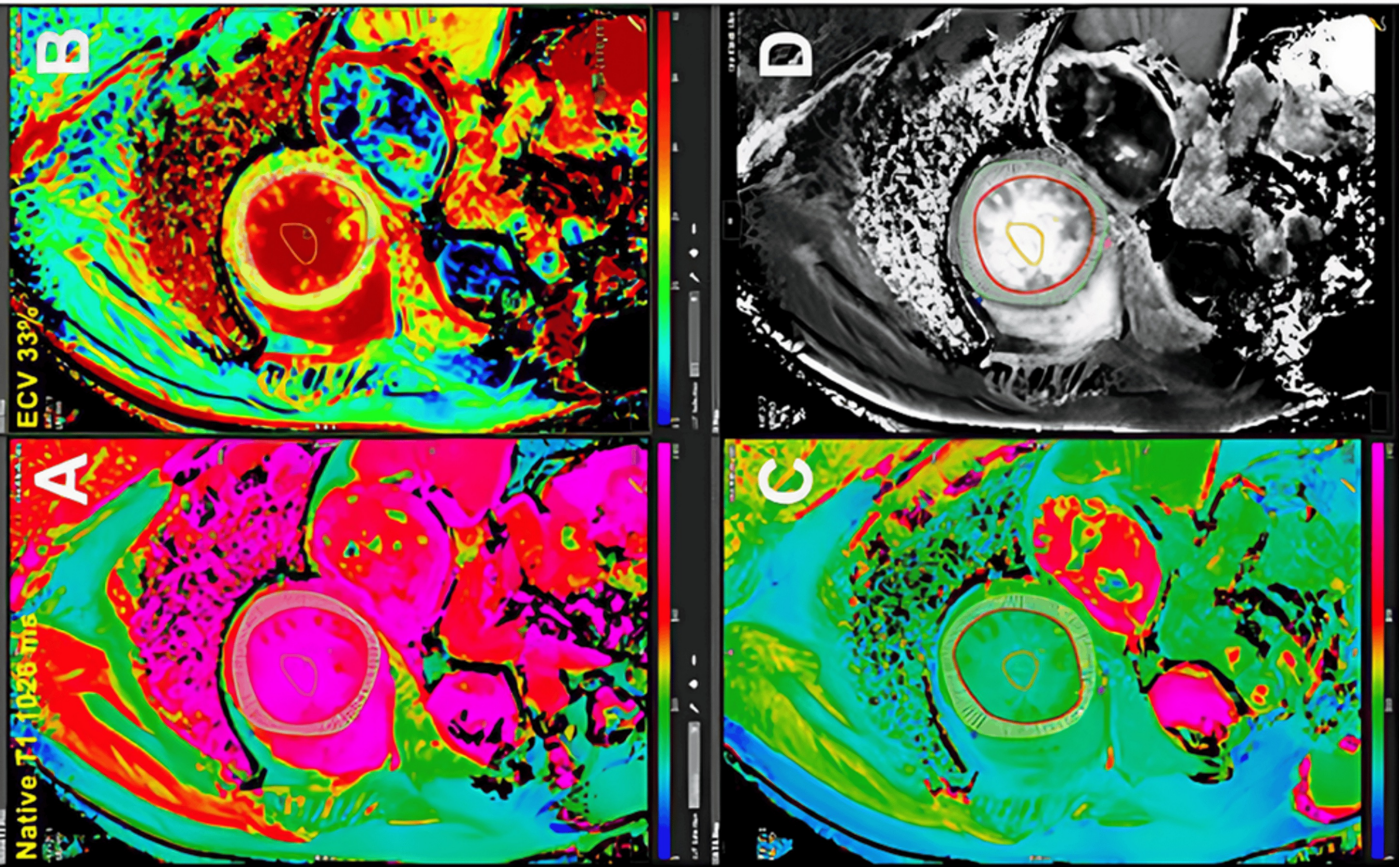
Myocardial T1 mapping and extracellular volume fraction assessment by cardiac magnetic resonance imaging. A) Native T1 mapping, acquired before contrast administration, reflects intrinsic myocardial properties and is useful for identifying diffuse abnormalities such as fibrosis, edema, or infiltrative processes. B) Extracellular volume fraction, a quantitative measure of the myocardial extracellular space derived from pre- and post-contrast T1 values in conjunction with hematocrit, allows assessment of diffuse fibrosis or infiltration. C) Post-contrast T1 mapping, obtained after gadolinium administration, demonstrates contrast distribution within the myocardium and serves as a basis for further quantitative analysis. D) Partition coefficient (λ) map, reflecting the relative distribution of gadolinium between blood and myocardial tissue, is commonly used as a surrogate marker for extracellular volume fraction. Figure reproduced from Gonska et al., licensed under CC BY 4.0 [25].

As illustrated in Figure 1, the combination of native and post-contrast T1 mapping enables the calculation of extracellular volume fraction, providing a quantitative estimate of diffuse myocardial fibrosis [25]. Post-contrast T1 mapping extends tissue characterization by incorporating gadolinium-based contrast agents, which distribute within the extracellular compartment. By combining pre- and post-contrast T1 values with hematocrit measurements, extracellular volume fraction can be calculated, providing a quantitative estimate of interstitial expansion. Extracellular volume fraction has demonstrated a strong correlation with histological collagen content, making it a reliable surrogate marker of diffuse myocardial fibrosis. However, its estimation is influenced by several methodological factors, including the accuracy of hematocrit measurement and the kinetics of contrast distribution, which may introduce variability in certain clinical settings [23,24,26].

Clinically, T1 mapping has substantial diagnostic and prognostic significance. Its ability to detect diffuse myocardial fibrosis at an early stage is particularly valuable in diseases such as dilated and hypertrophic cardiomyopathy, where early intervention may influence disease progression [26,27]. While late gadolinium enhancement remains effective for identifying focal replacement fibrosis, T1 mapping provides a more comprehensive assessment of myocardial involvement by capturing diffuse interstitial changes and offering incremental prognostic information [8,22]. Elevated native T1 values and increased extracellular volume fraction have been independently associated with adverse clinical outcomes, including heart failure progression and arrhythmic events, reinforcing their role as important biomarkers for risk stratification and longitudinal patient management [27-29].

Myocardial T2 mapping

**Figure 2:**
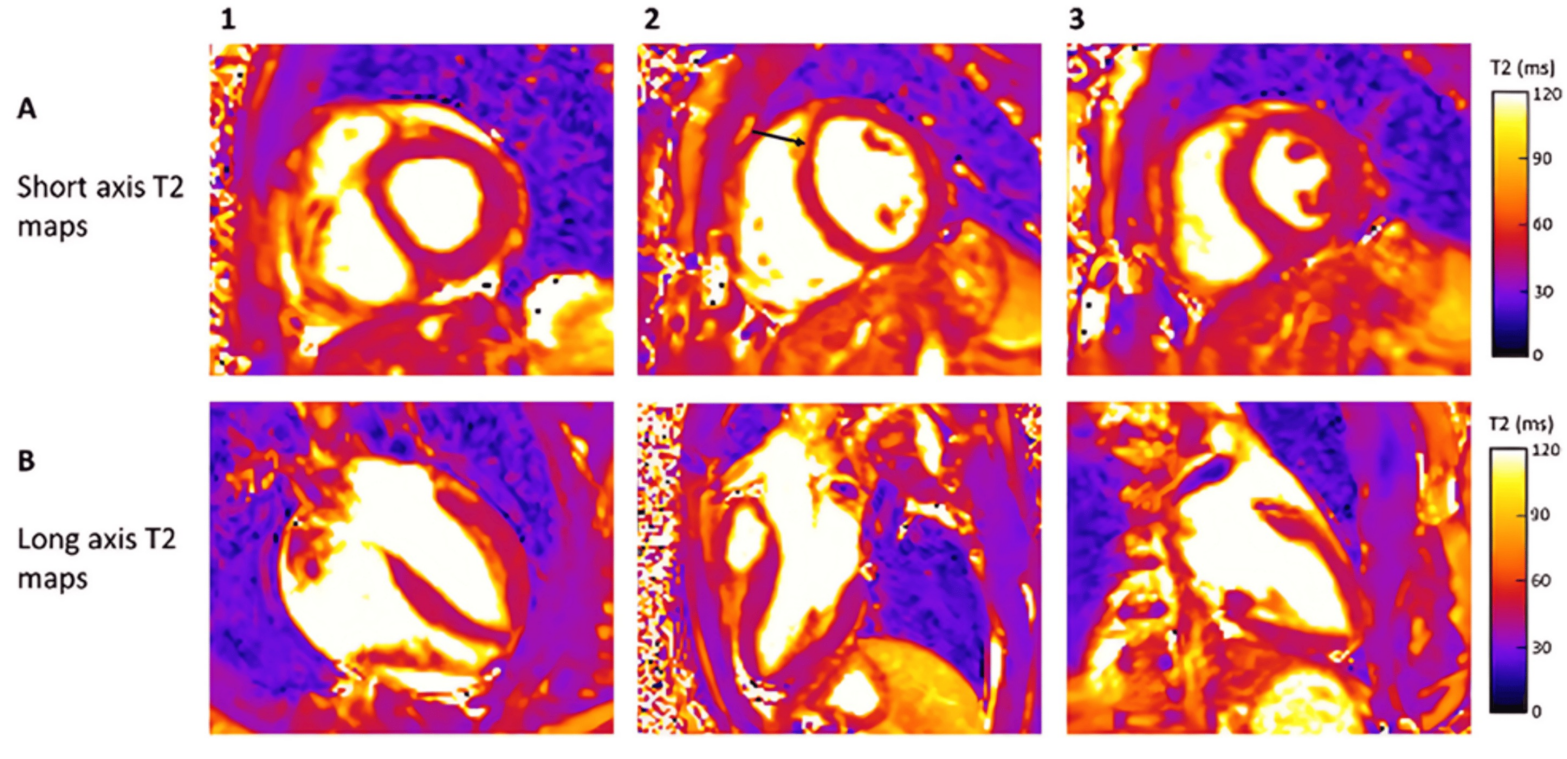
Representative T2 mapping images in a patient without evidence of myocardial edema or active inflammation. A) Short-axis views (A1-A3), where A1 corresponds to a basal short-axis view, A2 to a midmyocardial short-axis image acquired during diastole, and A3 to a midmyocardial short-axis image acquired during systole. A focal T2 prolongation in the mid anteroseptal wall is observed in A2 (black arrow), likely representing a partial volume artifact, which is no longer present in A3. B) Long-axis views (B1-B3), including B1 (horizontal long axis), B2 (three-chamber view), and B3 (vertical long axis), all demonstrate no abnormal T2 elevation. Figure reproduced from O’Brien et al., licensed under CC BY 4.0 [8].

The physiological basis of T2 relaxation is closely related to myocardial water content and to the decay of transverse magnetization caused by spin-spin interactions between neighboring hydrogen nuclei. When the proportion of free water within myocardial tissue increases, as occurs in pathological states such as edema and inflammation, T2 relaxation times become prolonged. Consequently, elevated T2 values are widely interpreted as quantitative markers of myocardial edema and serve as indirect indicators of active inflammatory processes affecting the myocardium. T2 mapping exploits this property by enabling direct quantification of myocardial edema in a non-invasive manner, and without the need for contrast agents, making it particularly suitable for repeated assessments and longitudinal follow-up [8]. An example of myocardial T2 mapping in the absence of edema or active inflammation - including the impact of acquisition phase and partial volume artifacts - is shown in Figure 2.

From a clinical perspective, T2 mapping has demonstrated high effectiveness in the detection of myocardial edema, which represents a hallmark of active inflammation. This technique is therefore commonly applied in conditions such as myocarditis and acute myocardial infarction, where inflammatory injury and tissue edema are prominent features of the disease process [8]. In addition, T2 mapping has shown high sensitivity for identifying myocardial inflammation in more specific clinical contexts, including myocarditis associated with systemic sclerosis. Its inclusion in the revised Lake Louise criteria has significantly improved diagnostic accuracy, underscoring its value as a key imaging biomarker of inflammatory myocardial involvement [30].

An important advantage of T2 mapping lies in its ability to differentiate between acute and chronic myocardial injury. Acute myocardial damage is typically characterized by edema and inflammatory infiltration, which manifest as elevated T2 values. In contrast, chronic fibrotic remodeling is generally associated with normalization of T2 relaxation times, while abnormalities are more prominently detected using T1 mapping and extracellular volume fraction measurements, which reflect collagen accumulation rather than active inflammation [1,30]. Notably, increases in T2 values often precede irreversible myocardial remodeling, allowing early identification of myocardial injury at a potentially reversible stage [8].

Despite its clinical utility, several technical factors may influence quantitative T2 measurements. Variations in magnetic field strength, differences in sequence selection, susceptibility effects, motion artifacts related to cardiac and respiratory motion, and partial volume contamination may affect the accuracy of T2 estimation. In addition, differences among scanner vendors and acquisition protocols may contribute to inter-scanner variability, particularly in multicenter studies. These factors highlight the importance of standardized acquisition protocols and appropriate reference ranges when interpreting T2 mapping results [23].

T2 mapping plays a complementary role alongside T1 mapping in comprehensive myocardial tissue characterization. While T2 mapping primarily reflects inflammatory activity and edema, T1 mapping is more closely associated with fibrosis and chronic structural alterations. The combined use of these techniques within cardiac magnetic resonance imaging enhances the ability to track the progression from acute inflammation to chronic fibrotic remodeling. This multiparametric approach provides a more complete understanding of disease evolution and supports improved clinical management and therapeutic decision-making in a wide range of cardiac diseases [6,31].

Clinical applications of T1 and T2 mapping

In ischemic heart disease, T1 and T2 mapping techniques have demonstrated significant clinical value for the assessment of both peri-infarct myocardium and remote myocardial remodeling. T1 mapping, in particular, allows differentiation among normal, ischemic, infarcted, and remote myocardial tissue by identifying distinct rest and stress T1 profiles. Stress T1 mapping has emerged as a promising contrast-free method for detecting myocardial ischemia, as ischemic myocardium demonstrates a blunted increase in T1 values during vasodilator stress, compared with normal myocardium. This capability enables detailed characterization of myocardial ischemia and infarction without the need for contrast administration, representing a major advantage for patients with contraindications to gadolinium-based contrast agents [32].

Beyond tissue characterization, parametric mapping plays a central role in risk stratification and prediction of ventricular remodeling following myocardial infarction. Both T1 and T2 mapping offer quantitative measures of myocardial fibrosis and edema, which are key determinants of post-infarction remodeling. Elevated native T1 values and increased extracellular volume fraction, often exceeding approximately 28%-30% in fibrotic myocardium, depending on acquisition parameters, have been associated with adverse ventricular remodeling and poorer clinical outcomes. Evidence indicates that extracellular volume fraction and infarct size, derived from mapping techniques, are strong predictors of left ventricular remodeling, functional deterioration, and subsequent cardiovascular events. These parameters, therefore, support improved risk stratification and inform clinical decision-making related to surveillance strategies and therapeutic interventions [33].

In the context of non-ischemic cardiomyopathies, T1 and T2 mapping techniques provide complementary insights into myocardial pathology. In dilated cardiomyopathy, T1 mapping is particularly useful for quantifying diffuse myocardial fibrosis, with elevated native T1 values often exceeding typical reference ranges of approximately 950-1050 milliseconds at 1.5 tesla, depending on the sequence used. These increases correlate with adverse clinical outcomes, such as progressive heart failure and ventricular arrhythmias. This quantitative assessment offers prognostic information that is not always captured by conventional imaging techniques. Similarly, in hypertrophic cardiomyopathy, combined T1 and T2 mapping facilitates detailed characterization of myocardial scarring, edema, and tissue heterogeneity, which is essential for risk stratification and individualized patient management [27,34].

T2 mapping is especially relevant in myocarditis and other inflammatory cardiomyopathies, where myocardial edema represents a hallmark of active disease. Quantitative T2 values, which typically range from approximately 45-55 milliseconds at 1.5 tesla in normal myocardium, are frequently elevated in the presence of active myocardial inflammation. By sensitively detecting increased myocardial water content, T2 mapping enables early identification of inflammatory involvement and allows longitudinal monitoring of disease activity. Importantly, this approach can be performed without contrast agents, making it suitable for repeated assessments and follow-up evaluations during the disease course [8].

Parametric mapping techniques also play an important role in infiltrative and systemic diseases affecting the myocardium. In conditions such as cardiac amyloidosis and sarcoidosis, T1 mapping and extracellular volume fraction often demonstrate markedly elevated values, reflecting expansion of the extracellular space. These techniques enhance the detection of myocardial involvement, support diagnostic accuracy, and assist in guiding management strategies by revealing diffuse myocardial changes that may not be evident with conventional imaging. Evidence from multicenter studies and clinical registries has further supported the prognostic value of mapping-derived parameters in these populations [35].

In valvular heart disease and states of chronic pressure overload, such as aortic stenosis and hypertensive heart disease, myocardial fibrosis represents a key determinant of clinical progression and outcomes. T1 mapping has been widely applied to quantify fibrotic remodeling in these conditions, offering objective measures that are critical for determining the optimal timing of intervention. The extent of myocardial fibrosis, assessed through mapping techniques, has been shown to correlate with postoperative recovery and long-term prognosis, thereby supporting its integration into preoperative evaluation [36].

The combined assessment of myocardial fibrosis and edema using T1 and T2 mapping has important implications for predicting postoperative outcomes and guiding clinical decision-making. Multiparametric cardiac magnetic resonance approaches that integrate T1 mapping, T2 mapping, and extracellular volume fraction provide a more comprehensive evaluation of myocardial remodeling. By quantifying myocardial tissue alterations before intervention, these techniques help anticipate functional recovery, risk of complications, and overall prognosis following surgical or interventional procedures. As such, parametric mapping contributes to a more personalized approach to patient management in valvular and pressure-overload heart diseases [37].

Comparison with other methods for fibrosis assessment

Late gadolinium enhancement imaging has long been considered a reference technique for the detection of myocardial scar and focal fibrosis, owing to its high spatial resolution and well-established clinical utility. However, this technique presents important limitations, particularly in the assessment of diffuse myocardial fibrosis, where contrast between normal and affected tissue may be insufficient for reliable detection. In addition, late gadolinium enhancement is inherently dependent on image quality and operator expertise, which can introduce variability in interpretation and limit reproducibility across centers [38]. In contrast, T1 and T2 mapping techniques provide quantitative pixel-wise assessment of myocardial tissue properties and are capable of identifying both focal and diffuse fibrotic changes. This broader tissue characterization allows for a more comprehensive evaluation of myocardial remodeling, and helps overcome several of the intrinsic constraints associated with late gadolinium enhancement imaging [1].

Echocardiographic strain analysis represents another non-invasive approach for the evaluation of myocardial fibrosis, primarily through the assessment of myocardial deformation and stiffness, which are known to be affected by fibrotic remodeling. By detecting subtle impairments in myocardial mechanics, strain imaging can identify early functional consequences of fibrosis before overt changes in global systolic function become apparent [39]. Despite these advantages and its widespread availability, strain analysis remains an indirect method of fibrosis assessment. It does not provide direct information on myocardial tissue composition and therefore lacks the ability to differentiate between fibrosis, edema, and other pathological substrates, with the same level of specificity achieved by cardiac magnetic resonance-based techniques [5].

Circulating biomarkers of myocardial remodeling have also been explored as tools for fibrosis assessment and risk stratification. These biomarkers can reflect underlying molecular and cellular processes involved in fibrotic pathways and may offer prognostic information in various cardiovascular diseases. Nevertheless, their diagnostic accuracy for detecting and quantifying myocardial fibrosis remains under investigation, and they are influenced by systemic factors that may limit their specificity for cardiac tissue [1]. In contrast, T1 and T2 mapping enable direct visualization and quantitative measurement of myocardial tissue changes, providing spatially resolved information on fibrosis and edema that circulating biomarkers cannot capture [40].

Beyond cardiac magnetic resonance and echocardiography, other imaging modalities have also been investigated for myocardial tissue characterization. Nuclear imaging techniques, including positron emission tomography using tracers targeting inflammatory or fibrotic pathways, have shown potential for identifying myocardial inflammation and remodeling processes. Similarly, advances in cardiac computed tomography have enabled the exploration of extracellular volume quantification and tissue characterization through dual-energy or contrast-enhanced techniques. Although these approaches remain less widely established than cardiac magnetic resonance mapping, they represent emerging, complementary strategies for evaluating myocardial pathology [33,39].

Despite their advantages, mapping techniques are not free from limitations. Quantitative T1 and T2 values may vary depending on scanner type, vendor-specific implementations, magnetic field strength, and acquisition protocols, which can affect comparability across institutions. These sources of variability highlight the importance of standardized acquisition techniques and locally validated reference ranges when interpreting mapping-derived parameters [2,38].

Overall, T1 and T2 mapping techniques offer several relative advantages compared with alternative methods. Their quantitative nature reduces operator dependency, facilitates longitudinal follow-up, and enables the detection of diffuse myocardial fibrosis that may be missed by late gadolinium enhancement imaging [1]. However, these techniques require specialized magnetic resonance equipment, standardized acquisition protocols, and experienced personnel for accurate interpretation, which may limit accessibility compared with more widely available modalities, such as echocardiography [29].

Prognostic and therapeutic implications

T1 mapping and extracellular volume fraction assessment have emerged as valuable tools for risk stratification, particularly in patients with nonischemic dilated cardiomyopathy. Elevated native T1 values and increased extracellular volume fraction reflect a greater burden of diffuse myocardial fibrosis, and have been associated with a higher risk of heart failure progression and arrhythmia-related events. As a result, these parameters function as independent predictors of major adverse cardiovascular events and provide prognostic information that complements traditional clinical and functional assessments [37]. In parallel, T2 mapping contributes to risk stratification by enabling the detection of myocardial edema, which represents an early marker of myocardial injury. This capability is especially relevant in conditions such as myocarditis and dilated cardiomyopathy, where the presence of edema may identify patients at increased risk during active or evolving stages of disease [8].

Beyond prognostic assessment, cardiac magnetic resonance imaging incorporating T1 and T2 mapping techniques plays an important role in monitoring therapeutic response. These quantitative imaging methods allow longitudinal evaluation of myocardial tissue composition and enable tracking of changes in fibrotic burden over time. Such monitoring provides objective insight into the effectiveness of antifibrotic and disease-modifying therapies, and supports a more precise assessment of treatment response. Given the dynamic nature of myocardial fibrosis and the heterogeneity of the cellular populations involved in the remodeling process, therapeutic strategies increasingly require individualized approaches. Quantitative cardiac magnetic resonance imaging contributes to this goal by providing reproducible and tissue-specific measurements that support personalized clinical management [6].

The incorporation of T1 mapping and extracellular volume fraction assessment into clinical decision-making algorithms further enhances their clinical relevance. These imaging biomarkers can improve identification of patients at high risk for malignant arrhythmias, and may assist in guiding decisions regarding advanced interventions, such as the implantation of cardioverter-defibrillator devices [38,41]. In addition, emerging research suggests that mapping-derived biomarkers may be integrated with clinical risk models, circulating biomarkers, and genetic markers to support multimodal risk stratification strategies. Furthermore, these quantitative imaging parameters may have implications for clinical trial design and therapeutic monitoring, particularly in the evaluation of emerging antifibrotic therapies targeting myocardial remodeling [40].

The ability of cardiac magnetic resonance imaging to non-invasively and quantitatively characterize myocardial tissue underpins its growing integration into clinical practice. By linking tissue characterization with prognostic assessment and treatment monitoring, parametric mapping techniques contribute to a more precise and individualized approach to cardiovascular care [1].

## Conclusions

Myocardial fibrosis represents a complex and heterogeneous pathological process, whose focal or diffuse distribution reflects distinct underlying mechanisms and clinical contexts. Its development is driven by tightly regulated cellular and molecular pathways, involving fibroblast activation, extracellular matrix accumulation, and inflammatory signaling, ultimately leading to myocardial stiffening, impaired cardiac function, and adverse ventricular remodeling. Although the prognostic impact of fibrosis varies according to the underlying etiology, it consistently correlates with disease severity, heart failure progression, arrhythmic risk, and mortality across both ischemic and non-ischemic cardiovascular diseases. Cardiac magnetic resonance imaging, with T1, T2, and extracellular volume mapping, provides a robust non-invasive framework for comprehensive myocardial tissue characterization. By capturing complementary information on fibrosis, edema, and inflammation, these parametric techniques enable differentiation between acute and chronic myocardial injury, and offer valuable insights into the continuum between inflammation and fibrotic remodeling.

Their quantitative nature helps overcome important limitations of conventional imaging modalities, particularly in the detection of diffuse myocardial fibrosis, and supports improved diagnostic accuracy, disease staging, and longitudinal monitoring. The integration of T1 and T2 mapping into clinical practice has important prognostic and therapeutic implications. These techniques enhance risk stratification, contribute to more informed timing of clinical interventions, support decisions regarding advanced therapies, and enable objective assessment of treatment response over time. In addition, ongoing technological developments, including artificial intelligence-assisted image analysis and automated mapping quantification, may further improve reproducibility, standardization, and clinical implementation of these techniques.
